# Non‐Equilibrium Fractionation Factors for D/H and ^18^O/^16^O During Oceanic Evaporation in the North‐West Atlantic Region

**DOI:** 10.1029/2022JD037076

**Published:** 2022-11-08

**Authors:** D. Zannoni, H. C. Steen‐Larsen, A. J. Peters, S. Wahl, H. Sodemann, A. E. Sveinbjörnsdóttir

**Affiliations:** ^1^ Geophysical Institute University of Bergen and Bjerknes Centre for Climate Research Bergen Norway; ^2^ Bermuda Institute of Ocean Sciences St. George’s Bermuda; ^3^ Institute of Earth Sciences University of Iceland Reykjavik Iceland

**Keywords:** water vapor, isotopes, nonequilibrium fractionation, evaporation, wind speed

## Abstract

Ocean isotopic evaporation models, such as the Craig‐Gordon model, rely on the description of nonequilibrium fractionation factors that are, in general, poorly constrained. To date, only a few gradient‐diffusion type measurements have been performed in ocean settings to test the validity of the commonly used parametrization of nonequilibrium isotopic fractionation during ocean evaporation. In this work, we present 6 months of water vapor isotopic observations collected from a meteorological tower located in the northwest Atlantic Ocean (Bermuda) with the objective of estimating nonequilibrium fractionation factors (*k*, ‰) for ocean evaporation and their wind speed dependency. The Keeling Plot method and Craig‐Gordon model combination were sensitive enough to resolve nonequilibrium fractionation factors during evaporation resulting into mean values of *k*_18_ = 5.2 ± 0.6‰ and *k*_2_ = 4.3 ± 3.4‰. Furthermore, we evaluate the relationship between *k* and 10‐m wind speed over the ocean. Such a relationship is expected from current evaporation theory and from laboratory experiments made in the 1970s, but observational evidence is lacking. We show that (a) in the observed wind speed range [0–10 m s^−1^], the sensitivity of *k* to wind speed is small, in the order of −0.2‰ m^−1^ s for *k*_18_, and (b) there is no empirical evidence for the presence of a discontinuity between smooth and rough wind speed regime during isotopic fractionation, as proposed in earlier studies. The water vapor *d*‐excess variability predicted under the closure assumption using the *k* values estimated in this study is in agreement with observations over the Atlantic Ocean.

## Key Points

- Observation‐based nonequilibrium fractionation factors for water isotopologues during ocean evaporation are proposed
- Significant correlation observed between nonequilibrium fractionation factors and 10‐m wind speed
- No evidence of distinction is found between smooth and rough regimes for isotopic fractionation during ocean evaporation

## 1 Introduction

Stable isotopes of hydrogen and oxygen in water have been used successfully for more than 50 years to study processes of the Earth’s water cycle. Specifically, using water stable isotopes allows atmospheric processes of the water cycle to be studied on time scales spanning the scale of turbulent eddies to glacial–interglacial time scales (Galewsky et al., 2016). Modulation of the water vapor isotopic composition, hereafter in delta notation, is linked to several physical processes occurring in the atmosphere involving phase change and turbulent mixing. On the one hand, isotope ratios of ^18^O/^16^O (δ^18^O) and D/H (δD) in precipitation are largely controlled by upstream precipitation during moisture transport and isotopic equilibrium effect during phase changes (Craig, 1961; Dansgaard, 1964; Rozanski et al., 1993). So‐called temperature and continental effects visible on isotopic composition of precipitation are also visible on tropospheric water vapor (Galewsky et al., 2016). On the other hand, the deviation from the linear relationship between δ^18^O and δD (i.e., *d*‐excess = δD − 8 × δ^18^O) in precipitation is controlled by nonequilibrium effects linked to evaporative conditions of moisture source areas (Craig & Gordon, 1965; Merlivat & Jouzel, 1979), by moisture recycling above the continents (Risi et al., 2013), as well as by subcloud droplet evaporation (Stewart, 1975) and cloud microphysics (Ciais & Jouzel, 1994). The *d*‐excess signal in surface water vapor at daily and subdaily time scale has been shown to be largely affected by local surface fluxes, advection, and exchange with the free atmosphere both over land (e.g., Aemisegger et al., 2014) and over the ocean (e.g., Benetti et al., 2014).

### 1.1 Magnitude and Control of Nonequilibrium Fractionation During Ocean Evaporation: Objectives of the Study

Isotopic fractionation can occur under two different conditions during water phase change in the hydrological cycle: equilibrium and nonequilibrium. While isotopic fractionation effects under equilibrium conditions above 0°C are well understood, nonequilibrium fractionation effects are still poorly constrained. During evaporation, a nonequilibrium process, the relative weight of molecular and turbulent diffusion controls the magnitude of nonequilibrium fractionation. The molecular diffusivity ratios for HD^16^O/H_2_^16^O and H_2_^18^O/H_2_^16^O in air are 0.9757 and 0.9727 (Merlivat, 1978). However, these need to be scaled in evaporation models because evaporation in an oceanic environment is not a pure molecular diffusion‐controlled process but also includes a turbulence component, that is not fractionating (Brutsaert, 1965, 1975; Craig & Gordon, 1965). To account for this turbulent component, a parametrization of nonequilibrium fractionation factors dependent on wind speed as the only independent variable has been proposed (Merlivat & Coantic, 1975). This parametrization, together with relative humidity (RH) and sea surface temperature (SST), was further used in Merlivat and Jouzel (1979) to model the variability of water vapor *d*‐excess under a global closure assumption (CA; i.e., of a closed water budget). However, several recent studies have questioned the assumed role of SST and wind speed on the controls of nonequilibrium fractionation based on water vapor *d*‐excess observations (Bonne et al., 2019; Pfahl & Sodemann, 2014; Pfahl & Wernli, 2008; Steen‐Larsen et al., 2014, 2015; Uemura et al., 2008). Other studies argued that water vapor *d*‐excess above the ocean surface may not be influenced solely by ocean surface evaporative conditions, namely RH and SST, but also by the coupling between the marine boundary layer (MBL) and the free troposphere (Benetti et al., 2018; Galewsky et al., 2022) as well as by air–sea interactions during cold and warm advection (Thurnherr et al., 2021). Consequently, it can be concluded that a substantial uncertainty exists on the magnitude of nonequilibrium fractionation during evaporation in real‐environmental conditions and, still, no agreement exists on the controls of nonequilibrium fractionation by SST and wind speed (e.g., Gonfiantini et al., 2020). This lack of consensus drives the following questions: which nonequilibrium fractionation factors are the most accurate to use during the evaporation process in the MBL? Is there empirical evidence for a dependency between nonequilibrium effects and wind speed in the oceanic environment? If a relationship between wind speed and nonequilibrium fractionation exists, is it captured by the established parametrizations by Merlivat and Jouzel (1979) which are based on wind tunnel experiments? These research questions will be addressed in this study by the following:

1. Estimating the nonequilibrium fractionation factors for δ^18^O and δD that best fit the observed isotopic composition of the evaporation flux from the ocean surface.
2. Test the validity of theoretical parametrization of the wind speed effect on nonequilibrium fractionation with observations of the isotopic composition of the evaporation flux over the ocean.

The impacts and limitations of the method applied for estimating the nonequilibrium fractionation factors and the isotopic composition of evaporation flux from the ocean surface will be discussed in detail, focusing on potential SST and ocean isotopic composition inhomogeneities in the study area. Furthermore, we will discuss the sensitivity of the linear relationship between *d*‐excess and RH normalized to SST (*h*_*s*_) under the CA by using the nonequilibrium fractionation factors estimated in this study and other available data sets of water vapor observations in the MBL.

### 1.2 Estimating the Isotopic Composition of the Evaporation Flux Using Near‐Surface Water Vapor Observations

**Figure 1:**
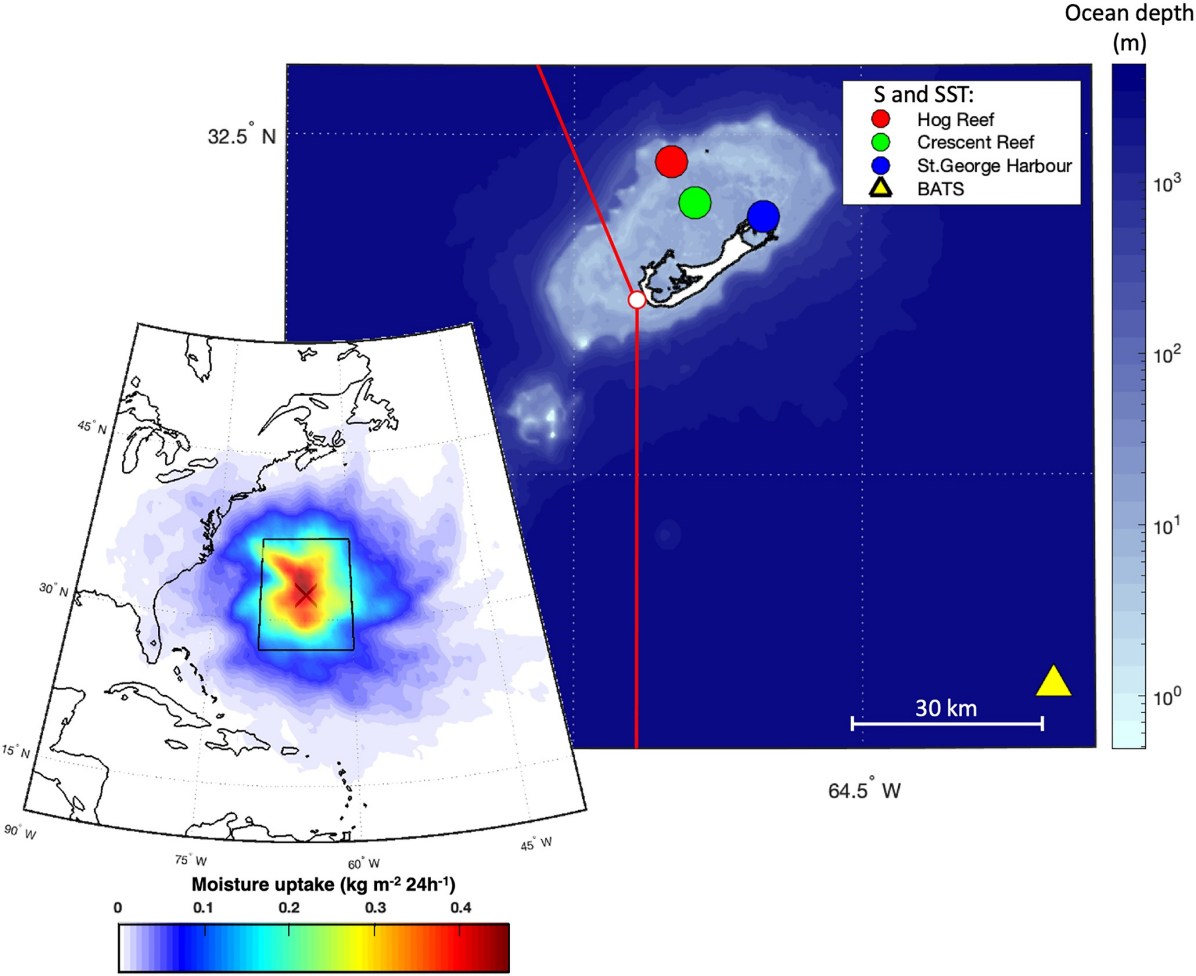
Study site in Bermuda. Bermuda Island shape in white color and ocean depth as color scale (Hijmans, 2015; NOAA, 2019): position of Tudor Hill Marine Atmospheric Observatory (white dot) and wind sector (red lines) to discriminate local transpired water vapor from ocean water vapor (N180° to N340°). Colored circles and triangle are the sampling locations of available salinity (S) and sea surface temperature (SST) time series around the study area. The large‐scale map on the left shows the location of Bermuda (cross) in the northwest Atlantic Ocean and the main water vapor sources during the study period. The highlighted sector includes 45% of accounted water vapor uptakes.

The isotopic composition of the evaporation flux can be estimated by three micrometeorological methods: Eddy Covariance (Braden‐Behrens et al., 2019; Wahl et al., 2021), Flux Gradient (FG, Yakir & Wang, 1996), and Keeling Plot (KP, Keeling, 1958). The Eddy Covariance method requires high‐frequency measurements of wind speed and vapor isotopic composition that are difficult to obtain but provides direct observations of the isotopic composition of the evaporation flux. FG and KP methods do not require high‐frequency measurements but rely on assumptions of the environmental conditions during evaporation. In principle, FG and KP can be used to estimate the isotopic composition of the evaporation flux by direct application of a fully turbulent mixing model between two end members: a constant water vapor flux and a background moisture of constant isotopic composition (binary mixing model). In this context, the application of KP and FG methods would be best addressed in an oceanic environment, where the main source of evaporating water is the ocean surface. Keeping the CA valid from a local point of view, that is, assuming that all the water vapor in the MBL originates from the evaporation flux, single level near‐surface observations of the water vapor *d*‐excess should be representative of local evaporative conditions, namely SST, RH, and wind speed. This assumption, however, is no longer valid when measurements are performed in low evaporation areas or for periods when other prevailing water vapor exchange processes, such as advection and/or entrainment, occur in the atmosphere. The expected wind speed effect could be smoothed out in the vapor *d*‐excess signal by other processes and observations of water vapor isotopic composition at a single height level then might not be representative of the evaporation flux. Furthermore, variability of the water vapor isotopic composition in the free atmosphere, during advection and via contribution of sea‐spray evaporation, can introduce errors in the estimation of the isotopic composition of the flux. Therefore, observations at different height levels should be used to estimate the isotopic composition of the evaporation flux with KP and FG instead of single height time series of water vapor isotopic composition. Many profile measurements are available in continental settings from atmospheric research and flux towers (e.g., Griffis et al., 2016) but are scarce over the ocean. Most of the available profile observations over the ocean were acquired over short time frames with cryotrapping (Craig & Gordon, 1965; Gat et al., 2003). More recently, two‐height profiles were obtained during research cruises but some additional uncertainties were introduced due to the use of different instruments for isotopic analysis at each height, ship movement, ship exhaust, and ocean spray contribution to the vapor composition (Thurnherr et al., 2020). In this study, we analyze a unique 6 months (20 June to 30 December 2013) data set of continuous observations of water vapor isotopic composition sampled at two heights from a meteorological tower located in the northwest Atlantic region (Bermuda, Figure 1) and use it to estimate the isotopic composition of the ocean evaporation flux.

Given that Bermuda is located in part of the source region for the precipitation which is deposited in Greenland, this study is also relevant for ice core science (Johnsen et al., 1989; Sodemann et al., 2008), questioning the type of information deduced from *d*‐excess in paleoclimate archives on the evaporative conditions at the source regions such as the role of wind speed and SST (e.g., Jouzel et al., 2007; Markle et al., 2018; Osman et al., 2021; Steen‐Larsen et al., 2011).

## 2 Materials and Methods

### 2.1 Study Site

The study site is located in the south‐western part of Bermuda, at the Tudor Hill Marine Atmospheric Observatory (THMAO) operated by Bermuda Institute of Ocean Sciences (32.26°N 64.88°W). The THMAO tower faces the coast (distance ∼30 m) and is 20.5 m high. Considering the altitude of the tower base (∼29 m AMSL), the top of the tower faces the ocean at a height of ∼50 m AMSL. The climatic conditions at Bermuda are characterized by a humid subtropical climate, strongly affected by the Gulf Stream. The study area is situated in the so‐called Bermuda‐Azores High, a large subtropical center of high atmospheric pressure. The high‐pressure system is primarily centered near the Bermuda Islands during summer and fall, and near Azores during winter and early spring. Ocean evaporation around Bermuda Island is strong due to its location near the Gulf Stream area and due to cold air advection, especially during the winter (Aemisegger & Papritz, 2018). ERA5 reanalysis data (Hersbach et al., 2020) show that the evaporation flux (E) in the study area exceeds the precipitation flux (P), as expected (P − E = −1.34 mm day^−1^). Analysis with a Lagrangian moisture source diagnostic (Läderach & Sodemann, 2016) for the June–December 2013 observation period calculated with ERA‐Interim reanalysis data (Dee et al., 2011) at a 1° × 1° resolution and a 6 hr time step revealed that 45% of lower tropospheric moisture originated in a 10° × 10° area around the study site (inset map in Figure 1). The evaporation flux footprint was also evaluated with a flux footprint model (Kljun et al., 2015), suggesting that 90% of the fetch area at the top of THMAO is within 2,800 m. Due to its position and climatic conditions, the island of Bermuda is therefore an ideal study site for evaporation‐related processes and their control on the *d*‐excess signal because ocean evaporation is the dominant source of the MBL vapor and there is low influence of continental water vapor.

### 2.2 Meteorological and Ocean Observations

Air temperature (T), RH (Campbell Scientific EE181‐L125‐PT), wind speed (WS), and wind direction (WD; R.M. Young CAT NO. 05103) were measured at the top inlet (50 m AMSL) of THMAO. The wind speed measured at 50 m AMSL was corrected to 10 m AMSL assuming a log‐law wind profile and a roughness length of 0.2 mm (Stull, 1997). Sea level pressure (SLP) and precipitation (P) were measured ∼20 km northeast at the L. F. Wade International Airport (TXKF) by the Bermuda Weather Service. MBL height data were retrieved from ERA5 global reanalysis data (blh variable), which is based on a critical value of the bulk Richardson number and depends on the vertical wind shear and buoyancy (ECMWF, 2017). Gridded blh was retrieved at 0.25° × 0.25° and 1‐hr temporal resolution and was linearly interpolated to the study site location.

### 2.3 SST and Ocean Water Isotopic Composition

Salinity and SST observations are available from buoys inside the reef at 3 hr time resolution (Hog Reef and Crescent Reef), at St. George Harbor at daily resolution, and outside the reef at monthly resolution for the Bermuda Atlantic Time‐series Study (BIOS, 2021). Salinity and SST measurement locations are reported in Figure 1. To minimize potential bias due to local SST variations, we chose the averaged Operational Sea Surface Temperature and Sea Ice Analysis (OSTIA, UK MET OFFICE, 2005) data as representative for SST of the study site. High correlation is observed between average SST measured inside the reef and OSTIA product averaged on a 1° × 1° box centered on Bermuda (*R* Pearson > 0.96) but better agreement, in terms of maximum absolute difference, was observed between BATS and OSTIA data (1.08°C) than for Crescent Reef and OSTIA data (2.55°C).

No measurements of ocean water isotopic composition near the study site are available for the period of interest, but the temporal variability of the ocean isotopic composition in the study area is assumed to be very low. Several sources have been evaluated for estimating the most representative composition of ocean water around the study site: gridded data set (LeGrande & Schmidt, 2006), North Atlantic cruises published data (Benetti, Reverdin, et al., 2017; Benetti et al., 2014) as well as from samples collected at the BATS site 2 years before this campaign (BIOS, 2021). The isotopic composition of the ocean in this study is assumed to be δ^18^O_L_ = 1.09‰ and δD_L_ = 7.25‰, which is the average between the isotopic composition calculated with the salinity to isotope conversion (Benetti, Reverdin, et al., 2017) applied to local salinity data (BIOS, 2021) and the ocean isotopic composition estimated from gridded data set (LeGrande & Schmidt, 2006). Full details on ocean water isotopic composition are reported in Text S1 in Supporting Information S1.

### 2.4 Water Vapor Isotopic Composition and Humidity Observations

Ambient air was sampled at THMAO tower at two different heights: 2.5 and 50 m AMSL. Ambient air was continuously pumped from the two inlets to a manifold located at the tower base that was connected to a Picarro L2120‐i isotopic water vapor Cavity Ring‐Down Spectroscopy (CRDS) analyzer. Quick air transport was ensured through heated copper tubing using a 10 L min^−1^ sampling pump. The sampling line was switched between the two inlets every 15 min and when one inlet was connected to the analyzer, the other inlet was flushed by a secondary 5 l min^−1^ pump. This configuration ensured a continuous circulation of air inside the tubing system, thus minimizing the lag and memory effect for the two inlets. The CRDS analyzer sampled water vapor from the main line at its nominal flow rate (∼40 sccm min^−1^) and recorded humidity and water isotopic composition at ∼0.56 Hz frequency. To reduce the memory effect due to the switching between top and bottom inlet, the first 10 min of data after valve switching was removed and the last 5 min was averaged. In this way, the 5 min average is assumed to be representative of the isotopic composition during measurement for each level, which yields one measurement point per half hour per level. The inlet can be approximated to a first‐order low pass filter with transfer function *H* = 1/(*τ* + 1), where *τ* is the time the system’s response need to reach 63% of the final value for a step change from zero initial condition (*τ*(δ^18^O) = 212 s, *τ*(δD) = 310s). Assuming the final value of the signal to be 1 for a normalized step change, we estimated that the magnitude (mag) of signal attenuation is only −1.9 dB for δ^18^O and −3.4 dB for δD (dB = 20 log_10_(mag)) and the phase difference between δ^18^O and δD signal is <9° with an averaging window of 0.5 hr. The error introduced by signal attenuation and phase difference between δ^18^O and δD signal in the system is considered insignificant at the time resolution used in this study. However, a small persistent bias in *d*‐excess can still be present during monotonically variations of δ^18^O and δD signals.

The isotope readings of the water vapor analyzer were calibrated on the VSMOW‐SLAP scale (IAEA, 2009) using several laboratory standards at the beginning and toward the end of the observation period. Drift‐correction measurements were carried out on a subdaily basis (every 6–12 hr) and humidity–isotope response curves were performed every 1–2 months during the study period to correct for the humidity dependency of water vapor isotopic composition. Precision of water vapor isotopic measurement is expected to be 0.14‰ for δ^18^O and 1.1‰ for δD. The reader is referred to a previous study conducted at THMAO for additional details on the setup of the sensing system, on the calibration protocol, and on sensing system performances (Steen‐Larsen et al., 2014). Humidity observations of the CRDS analyzer (moist mixing ratio, *w* [ppmv]) were calibrated against RH observations at the top inlet.

### 2.5 Estimation of the Evaporation Flux Isotopic Composition

The isotopic water vapor observations acquired with the CRDS analyzer represent the time‐averaged atmospheric moisture composition at a certain height above sea level. We used the KP method between the two inlets to estimate the isotopic composition of the water vapor flux (δ_E_). In the KP method, δ_E_ is assumed to be equal to the intercept of the linear best fit model between the isotopic composition of water vapor (δ^18^O or δD) and the inverse of humidity (1/*w*) at the two different height levels. The uncertainties for δ_E_ (*σ*_δE_) were calculated as a function of instrument precision, sample size, and atmospheric conditions (Good et al., 2012). However, in our case, the number of observations for each time step is equal to the degrees of freedom required to calculate the uncertainty associated with the flux composition. Therefore, observations were grouped on a daily basis and the error on flux composition was calculated when more than two observations were available. It is important to note that the computation of the flux composition with the KP method is valid only under the following assumptions:

1. The mixing process in the gradient measurement space is fully turbulent and does not introduce any fractionation: turbulent diffusion is the same for all isotopologs.
2. Water vapor flux is constant with height: the mixing ratio and water vapor isotopic composition vertical profiles are characterized by a monotonic trend.
3. Variability of water vapor isotopic signal is not significantly affected by advection or entrainment from the free troposphere during the acquisition of water vapor profiles.
4. Isotopic composition of source water is constant in the time interval considered.

Therefore, water vapor observations were filtered to fulfill the above mentioned assumptions, as further discussed in Sections 3.2 and 5.1. It is worth noting that the regression method used to calculate the isotopic composition of the evaporation flux can also impact the result, as recently shown in Hu et al. (2021). In this study, we used the ordinary least squares method to evaluate the KP intercept. According to Hu et al. (2021), the ordinary least squares method is more robust than for example, the Geometric Mean Regression method and should be comparable with the York Solution method under large fetch conditions. In this context, the isotopic composition of water vapor measured at the top inlet is assumed to be representative of water vapor in the MBL with a fetch area similar to the one estimated with the moisture diagnostic. However, because the large height difference between the bottom and top inlets results into different fetch areas, the water vapor isotopic composition at the bottom inlet was corrected (δ^18^O = +0.07‰ and δD = +0.75‰) accounting for the SST difference between open ocean SST and reef area SST, as further discussed in Section 5.2.

### 2.6 Estimation of Nonequilibrium Fractionation Factors

The Craig–Gordon (CG; Craig & Gordon, 1965) model was used to calculate δ_E_ (‰) from the ocean surface following the notation introduced in Merlivat and Jouzel (1979), as reported in Equation 1:

(1) δE=(1−k)αVL1+δL−hs1+δA1−hs−1

where *α*_*V*/*L*_ (<1) is the equilibrium fractionation factor between vapor and liquid (Horita & Wesolowski, 1994), *h*_*s*_ (1) is the RH measured at the top of the turbulently mixed sublayer relative to ocean surface temperature (OSTIA SST [K], averaged on a 1° × 1° box centered on Bermuda), *k* (1) is the nonequilibrium fractionation factor, δ_A_ is the isotopic composition of atmospheric moisture (1), and δ_L_ is the isotopic composition of the ocean water (1). The nonequilibrium fractionation factor *k* (reported in ‰ hereafter) is estimated from a direct comparison between the observed (KP) and modeled (CG) isotopic composition of the evaporation flux. For a given flux observation *i*, it is possible to calculate *m* different values of the flux composition with the CG model by varying the nonequilibrium fractionation factors within a certain range. The best *k* values are then calculated by error minimization between the modeled and observed evaporation flux composition for each pair of top and bottom inlet observations in the filtered data set. To estimate the average values of *k*, the inverse of the errors of the observed flux composition were used as the weights in the computation of the average. Populations of mean nonequilibrium fractionation factors *k* were estimated with bootstrapping, repeating the above sequence for 10^4^ times with random resampling. Additional details on how the nonequilibrium fractionation factors are calculated are reported in Text S2 and Text S3 in Supporting Information S1.

## 3 Data Description

### 3.1 Data Set

**Figure 2:**
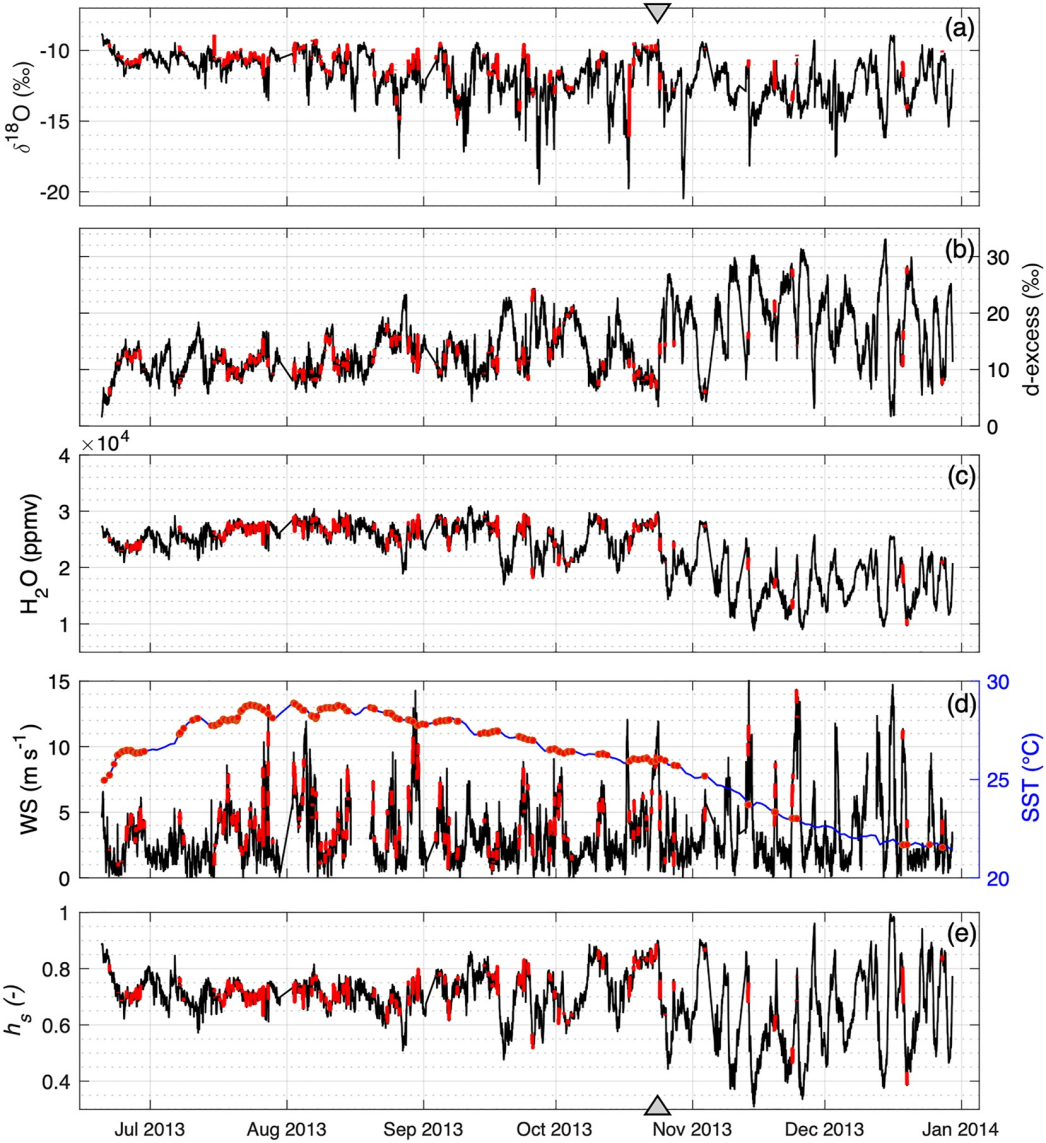
Time series of water vapor isotopic composition and relevant meteorological parameters at the study site. (a, b) Water vapor isotopic composition, (c) mixing ratio, and (d) wind speed (WS) measured at top inlet height (50 m AMSL). Sea surface temperature (SST) data from Operational Sea Surface Temperature and Sea Ice Analysis (OSTIA) reported as a blue line. (e) *h*_*s*_ is the relative humidity measured at top inlet and normalized to OSTIA SST. Gray triangles on the top and bottom of the figure represent the autumn transition, as detected from *d*‐excess variability at weekly scale. Observations selected for estimating the isotopic composition of the evaporation flux are highlighted in red.

Time series of water vapor at the top and bottom inlets were resampled using a common UTC time indexing with a resolution of 30 min through linear interpolation. Meteorological observations were also averaged and synchronized accordingly to CRDS observations. The water vapor time series used in this study includes 8,793 datapoints, representative of 30‐min averaged observations of water vapor isotopic composition at two height levels over the ocean surface. The complete data set accounts for 95% coverage of the study period (Figure 2).

Based on *d*‐excess, the pattern of atmospheric water vapor composition can be divided into two main groups: a first group from summer to midautumn with gentle daily to weekly *d*‐excess oscillations and a second group, from midautumn to early winter, with larger and more pronounced *d*‐excess oscillations at weekly scale. The transition in the *d*‐excess pattern follows the general decrease in humidity and the large *h*_*s*_ variability observed from late October (marked by gray triangles in Figure 2). The temperature decrease in autumn–winter is also linked to a small shift of the center of mass of moisture sources toward the north‐west (not shown). This shift can be linked to the increase in baroclinicity toward autumn and winter and to the more frequent passage of extratropical cyclones over the Gulf Stream leading to strong ocean evaporation (Aemisegger & Sjolte, 2018).

### 3.2 Filtered Data Set for Flux Estimation

To guarantee high data quality and for maximizing the validity of assumptions under KP (Section 2.5, points 1–4), several constraints were introduced to filter the data set. The rationale behind those constraints is summarized for each variable in Table 1. By means of the quality control filtering criteria, the sample size is reduced from 8,793 to 814 30‐min averaged observations (∼10% of available data). The variables that are most responsible for the exclusion of data points are the daytime and the western wind sector constraints. Just those two filtering criteria account for approximately 85% of rejections. However, these strict filtering criteria were necessary because of the local evapotranspiration signal contribution, with wind blowing from inland and dew formation caused by night cooling. The remaining filtering criteria accounted for an additional 5% of rejections.

**Table 1:** List of Variables and Constraints Adopted to Filter the Time Series

Variable	Indexing	Range/value	Rejected (cumulative; %)	Assumption	Rationale
				#	
Time	Time	Daytime observations based on sunrise–sunset hour (LST) with 2 hr symmetrical offset	71	2, 4	No influence of dew formation caused by night cooling
WD	Wind sector inclusion	Western sector	85	3, 4	No influence of local evapotranspiration from vegetation
		180°N–340°N (i.e., excluding winds from inland)			
δD and δ^18^O	|δD_Bottom_ − δD_Top_|	>1‰	89	2	Difference between Top/Bottom larger than instrumental precision (L2120‐i)
	|δ^18^O_Bottom_ − δ^18^O_Top_|	>0.1‰			
*w*	*w*_Bottom_ − *w*_Top_	>100 ppmv^a^	89	2	*w* decreases with height above ocean
P	Time	No precipitation within the last 2 hr	90	1–4	No vapor recycling from precipitation

*Note.* The column “rejected” reports the size of data set that does not fulfill each filtering threshold. Assumption *n*# refers to the numbered list in Section 2.5.

^a^ This is a conservative estimate of instrumental precision not reported in the L2120‐i datasheet.

Most of the observations (∼90%) of the filtered data set were selected between 20 June and 23 October, as shown in Figure 2. From the perspective of data representativeness, the main features of the data set after the filtering procedure are as follows: (a) slightly changed mean and median values (for δ^18^O and *d*‐excess) and reduction of secondary modes in *d*‐excess distribution; (b) statistically significant change in regression parameters for *d*‐excess versus *h*_*s*_; (c) significant reduction of observations characterized by deeper MBL (blh > 1,000 m, from 17% to 4%); (d) change of the wind speed distribution in terms of the mean (from 2.8 to 4.0 m s^−1^). Therefore, the main consequences of data reduction are a larger impact of shallow atmospheric mixing, a smaller influence of large MBL development, and less periods characterized by low wind speed conditions. More details on the impact of data filtering on the distribution shape of variables of interest are reported in Text S4 in Supporting Information S1.

## 4 Results

### 4.1 The Isotopic Composition of the Evaporation Flux (δ_E_) From the Ocean Surface

Descriptive statistics of the evaporation flux isotopic composition from the ocean surface and the water vapor isotopic composition observed at the top inlet during daytime are reported in Table 2. On average, the number of data points available for KP calculation is 20 per day and the coefficients of determination for both δ^18^O and δD regression lines are high (*R* ^2^ = 0.78, on average). For comparison, the FG method (Lee et al., 2007) was also used to compute the isotopic composition of evaporation flux, obtaining nearly identical results but different uncertainties, especially for δD (*σ*_δE_ = 0.59‰ and 51‰ for δ^18^O and δD, respectively). The high similarity between the FG and KP methods is consistent with other studies (Good et al., 2012; Hu et al., 2021) which is why we focused on the KP method. As expected, the isotopic composition of the flux is enriched with respect to the atmospheric water vapor composition and depleted compared to the ocean isotopic composition. The mean δD of the evaporation flux is between recent estimates of the global mean HDO fluxes (−37.6‰ following Good et al., 2015) and estimates made in past studies (−22‰ following e.g., Gat, 1996). No evident trend was observed for daily δ_E_ during the study period, for both δ^18^O and δD (Figure S6 in Supporting Information S1).

**Table 2:** Descriptive Statistics of Evaporation Flux and Top Inlet Water Vapor Isotopic Composition at the Daily Timescale

	Mean (‰)	Median (‰)	IQR (‰)	*σ*_δE_ (‰)
Evaporation flux δ^18^O	−3.37	−4.48	−6.7; −0.04	1.17
Evaporation flux δD	−24.99	−33.48	−48.38; −1.60	7.33
Top inlet water vapor δ^18^O	−11.30	−10.97	−12.10; −10.51	–
Top inlet water vapor δD	−78.13	−76.14	−83.15; −73.10	–

*Note.* Interquartile range (IQR) estimated by fitting a normal PDF on observed δ_E_ distribution. *σ*_δE_ following Good et al. (2012).

### 4.2 Nonequilibrium Fractionation Factor Distributions Estimated With Flux Observations

**Figure 3:**
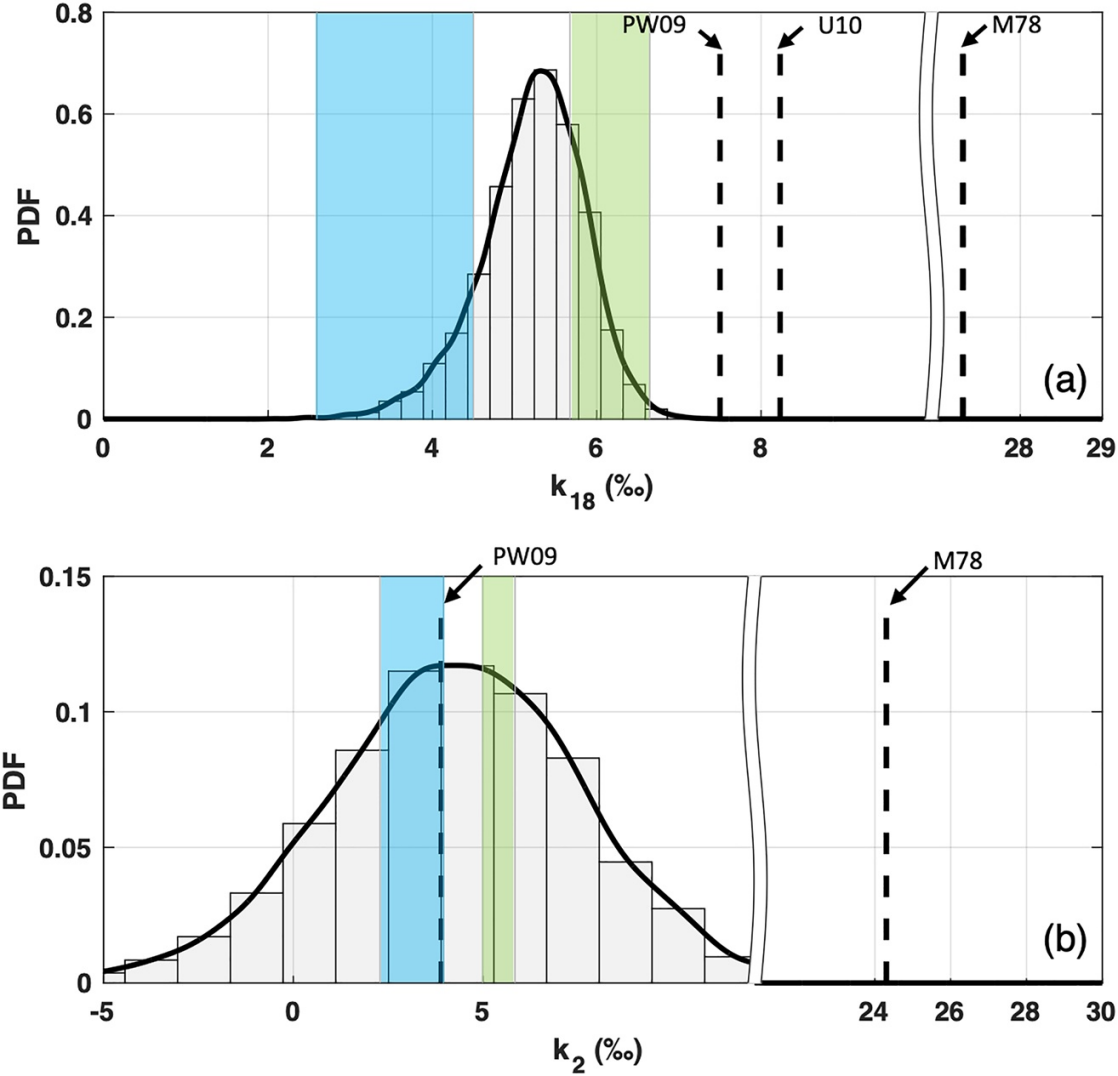
Nonequilibrium fractionation factors estimated from flux observations (Keeling Plot [KP] method). Continuous kernel density function was estimated with bandwidths 0.1‰ and 0.6‰ for *k*_18_ (a) and *k*_2_ (b), respectively. Shaded area represents the *k* intervals predicted for smooth (green, 10‐m wind speed range [1–6] m s^−1^) and rough (cyan, 10‐m wind speed range [6–13] m s^−1^) regimes following Merlivat and Jouzel (1979). For reference, molecular diffusivity ratios M78 (Merlivat, 1978) and nonequilibrium fractionation factors for ocean settings PW09 (Pfahl & Wernli, 2009) and U10 (Uemura et al., 2010) are reported as vertical dashed lines.

Nonequilibrium fractionation factors are expressed hereafter in term of *k*_18_ (for δ^18^O) and *k*_2_ (for δD) to allow a direct comparison with the parametrization proposed in Merlivat and Jouzel (1979). Applying the bootstrapping method (10^4^ samples with 80 observations in each) to the filtered data set yields a mean ± 1 std. dev., *k*_18_ = 5.21‰ ± 0.64‰, and *k*_2_ = 4.32‰ ± 3.41‰, as shown in Figure 3.

The obtained *k* PDFs are in the range predicted by the parametrization proposed in Merlivat and Jouzel (1979). For *k*_18_, the distribution of the mean values falls in the middle of the parametrizations for the smooth and rough wind speed regimes as proposed by Merlivat and Jouzel. A similar result was obtained for the average *k*_2_, the PDF of which is however characterized by a significantly larger spread. Consistent with previous works, nonequilibrium fractionation factors are on average ∼0.20–0.25 times the value expected for a purely diffusivity‐driven evaporation process (Merlivat, 1978). For reference, the *k* values estimated in other studies are also reported in Figure 3 (Pfahl & Wernli, 2009; Uemura et al., 2010). Note that the *k*_18_ values estimated in this study are 2‰–3‰ smaller than previous studies and more consistent with the parametrization of *k*_18_ proposed in Merlivat and Jouzel (1979). On average the ratio *k*_2_/*k*_18_ is equal to 0.83, similar to 0.88 in Merlivat and Jouzel (1979) and 0.84 reported in Luz et al. (2009).

### 4.3 Observed Relationship Between Nonequilibrium Fractionation Factors and 10‐m Wind Speed

**Figure 4:**
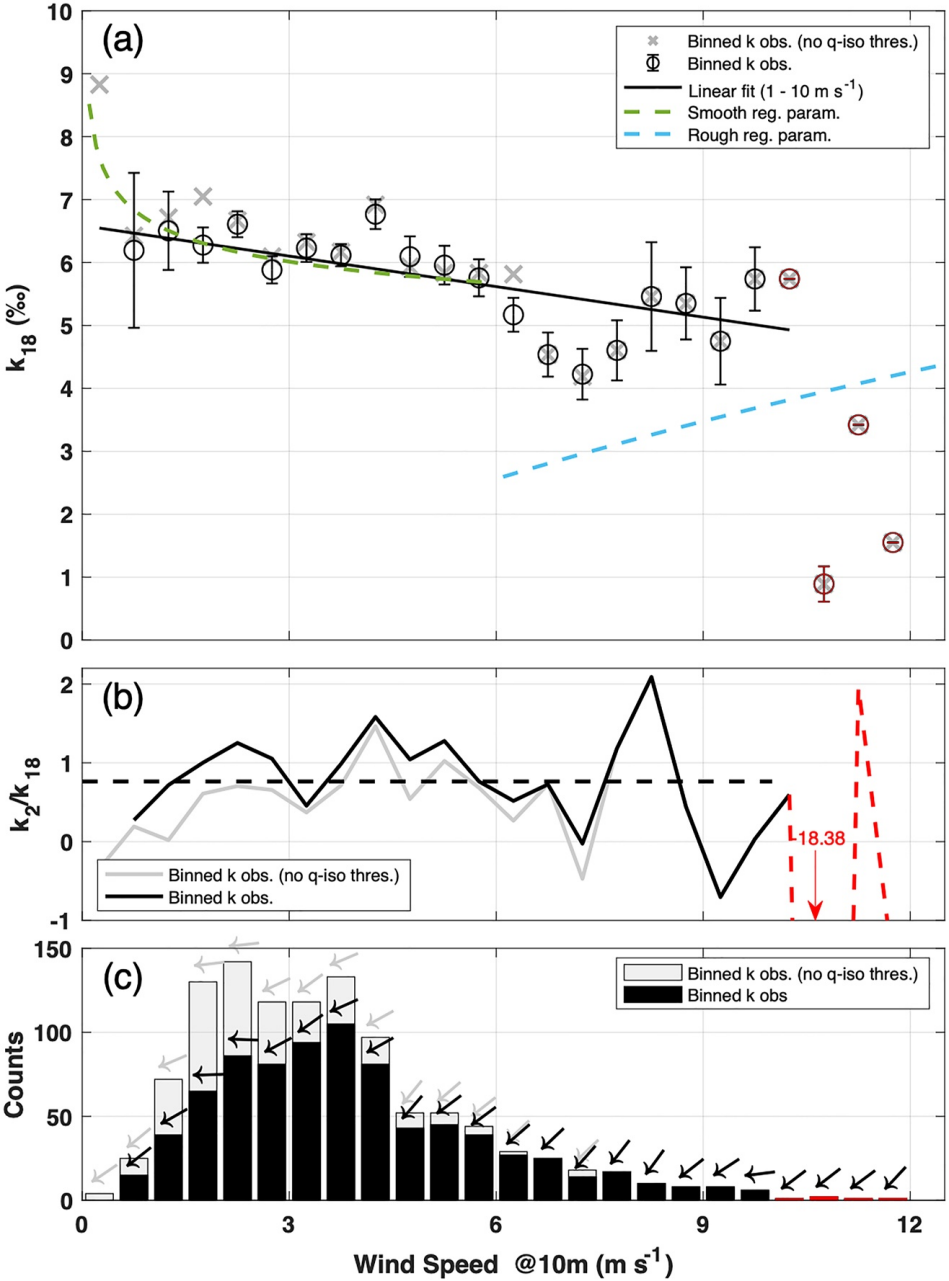
Observed relationship between *k*_18_ and 10‐m wind speed. (a) Mean ± standard error of *k*_18_ estimated for each wind speed class. Green and cyan lines show the parametrization of *k*_18_ for smooth and rough wind regimes, respectively (Merlivat & Jouzel, 1979). Solid black line represents a linear fit (*R* ^2^ = 0.52) in the wind speed interval 0.5–10 m s^−1^ (fit equation reported in text). (b) *k*_2_/*k*_18_ ratio for each wind speed class. Dashed black line is the average ratio (0.8). (c) Number of observations and mean wind direction (arrows) for each bin. In all panels: black lines, black symbols, and black bars for filtered data set; gray lines, gray symbols, and gray bars for filtered data set with no isotope and humidity thresholds implemented (Table 1, rows 3 and 4); red line, red symbols, and red bars highlight wind speed classes with number of observations ≤2.

To test a dependency of the fractionation factors *k* on wind speed, the filtered data set was binned in 10‐m wind speed classes with bin size 0.5 m s^−1^. For each wind speed class, the nonequilibrium fractionation factors were calculated using the KP method at 30 min time step. Afterward, mean and standard error of *k* were calculated for each wind speed bin center. Mean *k*_18_ values obtained in such way are reported as a function of 10‐m wind speed in Figure 4a.

In the wind speed range [0.5–10] m s^−1^, the negative correlation between *k*_18_ and wind speed is high and statistically significant (*r* = −0.72, *p*‐value = 1 × 10^−3^). The parametrization proposed in Merlivat and Jouzel (1979) agrees well with the observed *k*_18_ variability between 0.5 and 6 m s^−1^, with an average absolute difference of 0.1‰. Most importantly, the differences between parametrized and observed *k*_18_ values are normally distributed around zero (Kolmogorov–Smirnov and Shapiro–Wilk *p*‐values equal to 0.13 and 0.34, respectively) and the errors can therefore be attributed to random noise in the measurement. On the other hand, observed *k*_18_ are 2‰ larger than modeled *k*_18_ for rough regime parametrization between 6 and 10 m s^−1^. Moreover, the theoretical wide discontinuity between smooth and rough regime expected at ∼6 m s^−1^ is not visible in the observations. A decrease of *k*_18_ in the 7 ± 1 m s^−1^ wind speed region is noticeable but *k*_18_ observations quickly approach the main decreasing trend. The observed *k*_18_ values are on average 1.7‰ higher than the ones calculated with the rough regime parametrization between 7 and 10 m s^−1^. Despite the small number of observations at wind speed above 7 m s^−1^, this study (a) does not provide sufficient experimental evidence that there are two different regimes in the wind dependency of *k*_18_, and (b) suggests that a continuous decrease of *k*_18_ as a function of wind speed is more likely in the interval [0.5–10] m s^−1^. Such a decrease can be approximated by the following simplified equation:

(2) $$k_{18}=\left(-0.16\pm 0.04\right)\times \text{WS}+\left(6.6\pm 0.3\right)‰$$

where WS is the 10‐m wind speed in m s^−1^. Equation 2 highlights that in the wind speed range [0.5–10] m s^−1^ the sensitivity of *k*_18_ to wind speed is only −0.16‰ m^−1^ s ± 0.04‰ m^−1^ s. Data filtering prevents to calculate *k*_18_ at lower wind speed values, mainly because of the thresholds on humidity and isotopic composition differences between the two inlets. When such thresholds are removed, the number of observations increases on the left side of the wind speed distribution (Figure 4c), with a ∼5% increase of the sample size but yields a larger uncertainty for the lowest wind speed bin (SE = 1.7‰, not shown). The impact of the presence/absence of humidity and isotopic composition difference thresholds between the two inlets is minimal in the *k*_18_ wind speed relationship. Indeed, the average absolute difference of *k*_18_ with/without those thresholds is only 0.1‰ in the [0.5–10] m s^−1^ wind speed range, with a minimal increase of the slope of 0.04‰ m^−1^ s. Unfortunately, the limited number of datapoints above 10 m s^−1^ does not allow any other speculation on the dependency of *k*_18_ to higher wind speed and prevents a better constraining of the rough regime. Furthermore, it is possible that other processes such as sea‐spray contribution might start to become important in the net evaporation flux at higher wind speeds (Andreas et al., 1995; Veron, 2015). Therefore, Equation 2 must be considered valid only in the [0.5–10] m s^−1^ wind speed range.

Continuing with *k*_2_, observations are scattered and very noisy on the *k*_2_ versus wind speed coordinate plane (data reported in Figure S5 in Supporting Information S1) because δD is less strongly influenced by nonequilibrium fractionation than δ^18^O. The correlation between *k*_2_ and wind speed is low and not significant within the [0.5–10] m s^−1^ wind speed range (*r* = −0.34, *p*‐value = 0.15). Observations are not in agreement with Merlivat and Jouzel (1979) parametrization, neither for the smooth nor for the rough regime, with an average absolute difference of 1.4‰ from the model. The noise in *k*_2_ observations drastically affects the variability of the *k*_2_/*k*_18_ ratio, which shows an average value of 0.8 and a standard error of 0.1 (Figure 4b). It is worth noting that the *k*_2_/*k*_18_ ratio is not correlated with 10‐m wind speed.

## 5 Discussion

### 5.1 Method Sensitivity to Filtering Criteria

**Figure 5:**
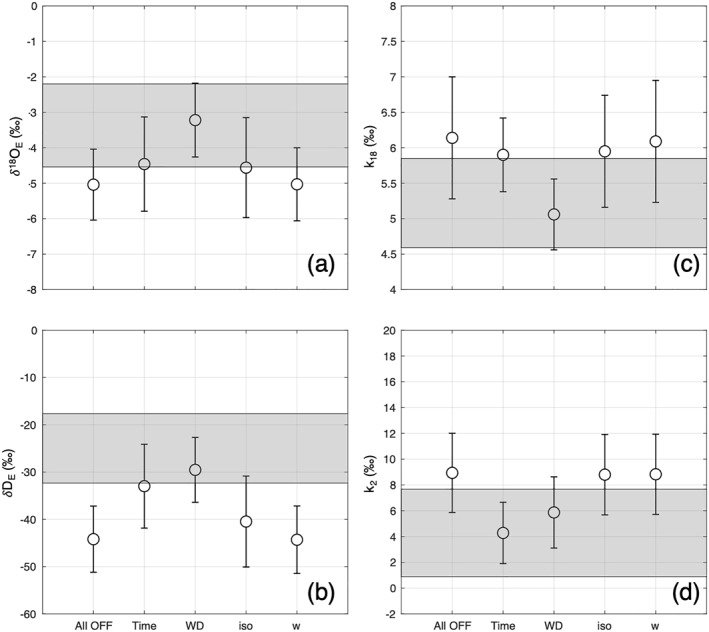
Sensitivity of the method for estimating δ_E_ and *k* values to filtering criteria. Following Table 1: only precipitation filter (All off, *n* = 6,834), time + precipitation filter (Time, *n* = 2,016), wind sector + precipitation (WD, *n* = 3,143), isotopic gradient + precipitation (iso, *n* = 3,883), humidity gradient + precipitation (*w*, *n* = 6,484). (a, b) Sensitivity of isotopic composition of evaporation flux (δ_E_) for δ^18^O and δD, respectively. (c, d) Sensitivity of nonequilibrium fractionation factors for *k*_18_ and *k*_2_, respectively. For all panels, gray shaded areas represent mean ± 1 std. deviation when enabling all filtering steps.

The KP method is based on assumptions that might partly be violated in a dynamic oceanic environment. Even on an island in the middle of the ocean, variability of local evaporation sources due to, for example, vegetation and change in wind direction can affect the validity of a simplified binary mixing model, with ocean and free atmosphere as the only end members. The strict filtering criteria used in this study to estimate the isotopic composition of the evaporation flux and the nonequilibrium fractionation factors try to select the data for maximizing the validity of the assumptions behind an ideal binary mixing model. This strict filtering, however, reduced the original data set size significantly, as mentioned before. Here, we discuss how each filtering criteria affects the results shown in Sections 4.1, 4.2, 4.3, removing only data that are affected by moisture input from precipitation events (Figure 5).

When all the filters are switched off, the isotopic composition of the evaporation flux decreases significantly and the mean δ_E_ values are not in accordance what would be expected for evaporation from the ocean (Craig & Gordon, 1965; Gat, 1996; Good et al., 2015), as shown in Figures 5a and 5b. Both daytime and western wind sector filters enrich the isotopic composition of the flux. However, westward wind direction has the largest impact on δ^18^O flux while daytime and westward wind direction filtering contributes likewise on δD flux. This different impact for δ^18^O and δD fluxes highlights the different sensitivity of the method to environmental changes in daytime–nighttime temperatures (larger effect on δD, minimal on δ^18^O) and on water vapor sources (ocean source vs. local evapotranspiration, similar effect for both δD and δ^18^O). The lower night temperatures, coupled to the poor ventilation due to low wind speeds during the night, increase RH substantially. Such stable conditions might promote the contribution of transpiration signal from local vegetation on the moisture near the ground. The *k* values show the mirror image of the evaporation flux composition. Indeed, wind direction filtering contributes the most on decreasing *k*_18_ value while time and wind direction contribute nearly equally to decreasing *k*_2_. Enabling/removing the thresholds on isotopic and humidity differences between the two inlets have only a marginal impact on the average flux composition and *k* estimation.

### 5.2 Impact of Ocean Surface Composition and SST Inhomogeneity in the Fetch Area on *k* Estimation

**Figure 6:**
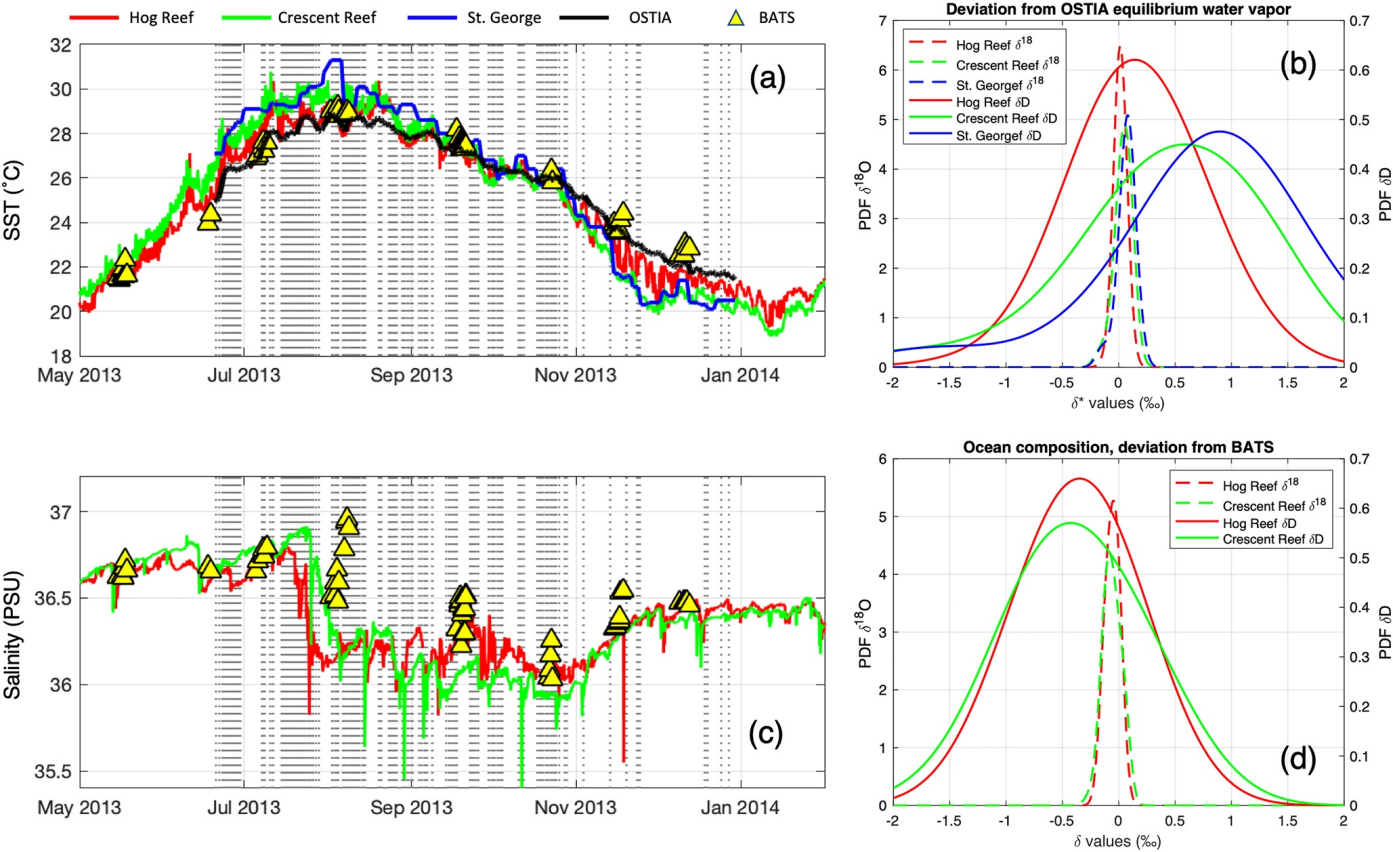
Sea surface temperature (SST) and salinity inhomogeneity of ocean waters around the study site. (a) Time series of SST in different points of the study area, see Figure 1 for reference of sampling sites. Vertical lines represent selected observations for flux estimation. (b) PDFs of δ^18^O and δD (equilibrium vapor [SST reef] − equilibrium vapor [SST OSTIA]), where SST reef is the SST measured in different points within the reef area. (c) Similar to (a) but for salinity. (d) PDFs of δ^18^O and δD (ocean composition [S reef) − ocean composition [S BATS]), where S reef is the salinity measured at different points within the reef area and S BATS is salinity measured at the BATS site. Conversion of salinity to isotopic composition following Benetti, Reverdin, et al. (2017).

The top and bottom inlets are sensitive to different fetch areas because of the height difference between the two inlets at THMAO (∼48 m). The flux footprint prediction model (Kljun et al., 2015) suggested that 90% of the fetch area for the bottom inlet is within 100 m while for the top inlet is within 2,800 m. The island of Bermuda is characterized by shallow waters close to the coast. Therefore, it is possible that local circulation of ocean water within the coral reef system can have an impact on SST variability and on surface water isotopic composition. Continuous measurement of SST and ocean isotopic composition covering the whole study area are not available. However, a first approximation of the variability of SST and salinity (as a proxy of evaporation) in the study area can be retrieved from buoys and BATS data, as shown in Figures 6a and 6c. In this context, the variability of SST can be used to estimate the equilibrium water vapor variability in the study area (Figure 6b), while the variability of salinity can be used to estimate the variability of ocean composition by applying the salinity to isotope conversion following Benetti, Reverdin, et al. (2017) (Figure 6d).

It is reasonable to assume that OSTIA SST is more representative of the isotopic composition of equilibrium water vapor for the top inlet, while the SST measured near the island coastline is representative for the bottom inlet. To account for the different fetch areas, we correct the water vapor isotopic composition at the bottom inlet by adding the mean deviation of St. George equilibrium SST from OSTIA, that is +0.07‰ and +0.75‰ for δ^18^O and δD, respectively (i.e., the mean of blue PDF in Figure 6b). As anticipated in Section 2.5, we used this correction to calculate the isotopic composition of evaporation fluxes shown in this study. Similarly, the ocean composition within the reef is likely more representative of evaporating water within the reef, hence, an approximate offset can be added to the isotopic composition of the ocean equal to −0.06‰ and −0.38‰ for δ^18^O and δD, respectively (i.e., the mean of the green PDF in Figure 6d). Next, we discuss how large is the impact of such corrections on the estimation of the *k* values and on the relationship between *k* and wind speed. As shown in Table 3, the inhomogeneity of ocean composition can introduce a bias in *k*_18_ and *k*_2_ in the order of 0.3‰ and 1.3‰, respectively. These biases are smaller than the uncertainties of *k*_18_ and *k*_2_. On the other hand, SST inhomogeneity in the study area can introduce a 0.8‰ and 8‰ bias in *k*_18_ and *k*_2_ estimation. When the SST correction is implemented, the *k*_18_ bias is still comparable to *k*_18_ uncertainty, while *k*_2_ differs significantly when the offset is introduced (66% absolute deviation). Therefore, SST has a larger impact on δD than on δ^18^O and the impact of ocean composition inhomogeneity in the study area is lower than the impact of SST in the estimation of *k*_18_ and *k*_2_. It should be noted that neither the correction for SST nor the correction for ocean composition take into account the magnitude of the evaporation flux in the estimation of the average δ_E_. The average δ_E_ should be in principle weighted by mass flux from the ocean surface. However, similar nonequilibrium fractionation factors were obtained with an SST correction based on the day‐by‐day difference between OSTIA and St. George SST instead of the mean difference during the whole study period (5.37 and 5.16 for *k*_18_ and *k*_2_, respectively). A key point is that SST correction and salinity + SST corrections lower the *k*_2_/*k*_18_ ratio below unity (0.83 and 0.57), which makes sense from the physical point of view, since the (1‐HD^16^O/H_2_^16^O) quantity needs to be smaller than (1‐H_2_^18^O/H_2_^16^O; e.g., as recently shown in Hellmann & Harvey, 2020). However, when both salinity and SST corrections are implemented, *k*_2_ is too low and not consistent, for example, with recent water vapor observations in the Atlantic Ocean (Bonne et al., 2019). Finally, both corrections do not significantly affect the observed correlation between *k*_18_ and wind speed. The main effect of the corrections on *k*_18_ and *k*_2_ leads to shifts in the distributions without changing their shapes. This means that the effect introduced by the correction is translated into changing the intercept of the best fit line of Figure 4a but keeping the slope mostly unchanged. The observed negative correlation between *k*_18_ and wind speed is robust, regardless of the correction implemented.

**Table 3:** Impact of SST and Ocean Composition Variability on *k*_2_, *k*_18_, and on *k*_18_ Versus Wind Speed Parameters Estimation

Correction	Cause	*k*_18_ (‰)	Dev. (%)	*k*_2_ (‰)	Dev. (%)	*k*_2_/*k*_18_	Slope (‰ m^−1^ s)	Interc. (‰)
No correction	–	6.0	–	12.74	–	2.11	−0.20	7.93
Salinity correction	Different isotopic composition of surface water in fetch area	5.8	−4	11.37	−11	1.96	−0.21	7.72
SST correction	SST inhomogeneity in fetch area	5.2	−14	4.32	−66	0.83	−0.16	6.59
Salinity + SST corrections	SST and surface composition inhomogeneity	5.0	−18	2.81	−78	0.57	−0.17	6.37

*Note.* Uncertainties are 0.6‰, 3.5‰, 0.04, and 0.3 for *k*_18_, *k*_2_, slope, and intercept, respectively. Deviations from *k* values obtained without applying any correction.

### 5.3 Suggested *k* Values and Limitations of the Approach

The large footprint difference for the two inlets is the highest source of uncertainty and the limitation in our experimental setup, even with the strict filtering criteria applied to the data set. The good agreement of our results with previous studies of the evaporation flux isotopic composition and the expected *k*_2_/*k*_18_ ratio in the expected range cannot serve as validation of our method, but they provide a constraint on identifying the highest uncertainty source. As outlined in Section 5.2, we identified SST differences in the footprint areas to be the main driver for the systematic bias observed for *k*_2_. Given that SST correction does not affect *k*_18_ significantly, we suggest using the mean value of *k*_18_ = 5.2‰ and *k*_2_ = 4.3‰. Indeed, these *k* values are estimated using all the observations that maximized the validity of KP method assumptions and thus should be representative for the average conditions. When simulating ocean evaporation in isotope‐enabled General Circulation Models, *k*_18_ can be calculated from the 10‐m wind speed using the empirical linear relationship (Equation 2) and *k*_2_ can be estimated by the average observed ratio of *k*_2_/*k*_18_ = 0.83. These values are valid for wind speed between 0.5 and 10 m s^−1^.

### 5.4 *d*‐Excess Sensitivity to Evaporative Conditions Using Suggested *k* Values

Assuming that the water vapor *d*‐excess signal is only modulated by local evaporation, the suggested nonequilibrium fractionation factors of this study can be used to predict water vapor *d*‐excess (‰) using *h*_*s*_ (%) and the CA. Table 4 reports the regression coefficients (slope and intercept) of the observed and modeled *d*‐excess versus *h*_*s*_ relationship using the data of this study and the data of four research cruises (Benetti, Steen‐Larsen, et al., 2017) which crossed the Atlantic Ocean between 2012 and 2015 at different latitudes (plots of *d*‐excess vs. *h*_*s*_ reported in Figures S3 and S7 in Supporting Information S1). For computation of *d*‐excess under CA, ocean δ^18^O was obtained from the LeGrande and Schmidt (2006) gridded data set, by averaging the closest four grid points of the ship location for each cruise, and ocean δD was estimated from the δ^18^O versus δD relationships (Benetti, Reverdin, et al., 2017). The slope of the modeled *d*‐excess versus *h*_*s*_ relationship is fully comparable with the one calculated for STRASSE cruise only. In general, the mean absolute error (MAE) and root means squared error (RMSE) increase as a function of the latitude for cruises, with negligible errors for PIRATA and STRASSE. When screening the Bermuda data set as shown in Section 3.2, the CA yields smaller regression coefficients in absolute values (−0.46‰/% and 46‰ for slope and intercept, respectively). A further decrease can be observed when the data set is screened also by removing observations with MBL height for example, larger than 1,000 m (−0.39‰/% and 40‰). Given that regression coefficients for Bermuda tend to agree with the one predicted under the CA and that the STRASSE cruise was characterized by shallow boundary layer (Benetti et al., 2014), atmospheric mixing between the MBL and the free atmosphere can be one of the processes causing the discrepancy between observed *d*‐excess variability in the MBL and the CA. Indeed, such a process (a) promotes the variability of the isotopic composition of water vapor in the free atmosphere and (b) modulates *h*_*s*_ in the MBL at the same time (Benetti et al., 2018; Risi et al., 2019). Although an input of water vapor from the free atmosphere violates assumption #3 in the KP method to calculate δ_E_ (see Section 2.5), we do not observe a significant change in estimation of δ_E_ and *k* values when screening also for MBL height (δ^18^O_E_ = −3.08‰ and δD_E_ = −23.06‰; *k*_18_ = 5.16‰ and *k*_2_ = 4.08‰). A regression model based on observed *d*‐excess, CA, and MBL height is able to reproduce 82% of the *d*‐excess signal variability in the entire Bermuda data set, showing that the 55% of variability can be attributed to *h*_*s*_ variability and 22% to MBL height variability. Although this simplified analysis considers *h*_*s*_ and the height of the MBL as two independent quantities, even though they are correlated, it shows that *d*‐excess signal in MBL water vapor might contain more information than evaporative conditions over the ocean surface. We therefore expect this study to highlight the need for more research effort to determine the processes driving *d*‐excess signal in the MBL at the daily − subdaily scale.

**Table 4:** *d*‐Excess (‰) Versus *h*_*s*_ (%) Relationship: Observed and Modeled Under CA

Data set	SST source	Observed *d*‐excess		Modeled *d*‐excess		MAE (‰)	RMSE (‰)
		Slope (‰/%)	Interc. (‰)	Slope (‰/%)	Interc. (‰)		
ACTIV (*n* = 3,087)	OSTIA (200 km × 200 km)	−0.32 (±0.01)	33.71 (±0.46)	−0.40	34.43 (±0.07)	6.52	3.47
RARA (*n* = 5,115)	On board SBE38 (1.50 m depth)	−0.38 (<0.01)	39.58 (±0.20)	−0.43	41.58 (±0.23)	2.60	2.46
STRASSE (*n* = 2,224)	On board SBE35 (3.50 m depth)	−0.38 (±0.01)	38.35 (±0.40)	−0.38	38.51 (±0.12)	1.12	1.44
PIRATA (*n* = 2,662)	On board SBE3S (3.33 m depth)	−0.24 (±0.01)	29.37 (±0.47)	−0.41	40.83 (±0.18)	0.89	0.93
Bermuda this study (*n* = 8,791)	OSTIA (1° × 1°)	−0.48 (<0.01)	47.91 (±0.16)	−0.36	35.48 (±0.04)	3.82	4.46

*Note*. Observations from ACTIV, RARA, STRASSE, and PIRATA cruises averaged over 15 min (Benetti, Steen‐Larsen, et al., 2017). Slopes and intercepts reported with their (±standard error). For modeled *d*‐excess, standard error of the slope is always <0.01. Mean absolute error (MAE) and root means squared error (RMSE) of observed *d*‐excess versus modeled *d*‐excess (CA).

## 6 Conclusions

Profile observations of water vapor isotopic composition near the ocean surface can be used to quantify the impact of nonequilibrium effects on isotopic fractionation during oceanic evaporation. In this study, we provided a unique data set of water vapor isotope observations collected at two different heights on a meteorological tower in Bermuda, located in the North Atlantic Ocean. Using the combination of the KP method and the CG model, we have calculated the nonequilibrium fractionation factors for ^18^O/^16^O and D/H during ocean evaporation and investigated their dependency on wind speed. A strict data filtering approach was used to maximize the validity of the assumptions behind the KP method, ensuring a robust estimate of the nonequilibrium fractionation factors. The observed nonequilibrium fractionation factor for ^18^O/^16^O is in good agreement with the established smooth wind speed parametrization in Merlivat and Jouzel (1979) (mean ± 1 std. dev., *k*_18_ = 5.2‰ ± 0.6‰). We find a statistically significant correlation between *k*_18_ and 10‐m wind speed, with a sensitivity in the order of −0.16‰ m^−1^ s to −0.20‰ m^−1^ s. Such low sensitivity would be nearly impossible to resolve by conventional measurements of the isotopic composition of water vapor at a single height above the ocean surface. Although the number of observations in high wind speed conditions is sparse in the observational data set, the observed relationship between *k*_18_ and wind speed does not provide a clear indication for the presence of a discontinuity between a smooth and rough surface under different wind regimes. In fact, the rough regime parametrization of *k*_18_ underestimates the observed fractionation factor by a factor of ∼0.66. Mean nonequilibrium fractionation factor for D/H was shown to be in the range expected following Merlivat and Jouzel (1979) albeit with a larger uncertainty (mean ± 1 std. dev., *k*_2_ = 4.3 ± 3.4‰). We showed that the spatial inhomogeneity of SST and ocean isotopic composition around the study site have an impact on the estimation of *k*_2_ and its uncertainty because of the large height difference between the two inlets and the resulting different fetch areas. The results for *k*_18_ are robust regardless of different data filtering and are insensitive to footprint correction based on the spatial variability of SST and ocean composition. Lastly, using the nonequilibrium fractionation factors of this study and the CA, we showed that the *d*‐excess signal in water vapor at the daily − subdaily temporal scale over the ocean contains information on MBL height in addition to SST and RH. The results of this study allow more accurate simulation of *d*‐excess in the MBL, hence allowing observations to be used to improve the fidelity of isotope‐enabled numerical models when simulating ocean evaporation.

## Supporting information

Supporting Information S1

## Data Availability

The water vapor time series used for calculating the nonequilibrium fractionation factors in the study is available in PANGAEA, DOI to be minted with CC BY 4.0 (Steen‐Larsen et al., 2022). Code for data analysis and for reproducing plots in the article is available in Zenodo (Zannoni, 2022).
