# Supplementary material for: Non‐Equilibrium Fractionation Factors for D/H and 18O/16O During Oceanic Evaporation in the North‐West Atlantic Region
Source: J Geophys Res Atmos. 2022 Nov 8;127(21):e2022JD037076. doi: 10.1029/2022JD037076 (PMC9786641; doi:10.1029/2022JD037076)
Supplement: Supplementary file 1 — Supporting Information S1 [file JGRD-127-e2022JD037076-s001.pdf]

Journal of Geophysical Research: Atmospheres

Supporting Information for

Non-Equilibrium Fractionation Factors for D/H and $^{18}O/^{16}O$ During Oceanic Evaporation in the North-West Atlantic Region

D. Zannoni[1], H. C. Steen-Larsen[1], A. J. Peters[2], S. Wahl[1], H. Sodemann[1] and A. E. Sveinbjörnsdóttir[3]

[1]Geophysical Institute, University of Bergen and Bjerknes Centre for Climate Research, 5007, Bergen, NORWAY.

[2]Bermuda Institute of Ocean Sciences, St. George's GE01, BERMUDA.

[3]Institute of Earth Sciences, University of Iceland, Reykjavik, ICELAND.

Contents of this file

Text S1 to S4
Figures S1 to S7
Tables S1

Introduction

In this Supporting Information file are reported all the additional explanations, table and figures regarding methods and results that are not critical for the outcome of the main manuscript but might help providing a clearer picture of the study to the reader. In order, the supporting file includes:

Text S1, Table S1: Values and references used to estimate the isotopic composition of the ocean around Bermuda

Text S2, Figure S1: Explanation of the Craig Gordon model focusing on non-equilibrium fractionation factors.

Text S3: Explanation of the algorithm used to estimate non-equilibrium fractionation factors

Text S4, Figures S2 – S4: Effect of data filtering on PDFs of main variables

Figure S5: scatterplot of k_2 vs 10-m wind speed

Figure S6: timeseries of $\delta^{18}O_E$ and δD_E.

Figure S7: Water vapor d-excess vs h_s observed (Benetti et al. 2017b) and predicted using k_2 and k_{18} of this study.

Text S1: Isotopic composition of the ocean.

No measurements of ocean water isotopic composition near the study site are available for the time period of interest. However, the temporal variability of ocean isotopic composition in the study area is expected to be very low. Several sources have been considered to estimate the most representative composition of ocean water around the study site: gridded dataset, North Atlantic cruises published data as well as from samples collected at the Bermuda Atlantic Time-series Study (BATS) site during 2012. Details for these datasets are briefly reported in Table S1. $\delta^{18}O$, δD and S data from BATS cruises for summer 2012 are fully comparable with Western North Atlantic Ocean data for summer 2015 and 2016 around Bermuda area and with Eastern North Atlantic Ocean data for summer 2012. All $\delta^{18}O$ observations reported in Table S1 are more enriched than in gridded dataset[3], but still comparable when considering uncertainties. We calculated the isotopic composition of ocean water for this study by applying the δ vs S relationship (Benetti et al., 2017a) and combining salinity measurements performed during the study period (Hog Reef, Crescent Reef and BATS) and gridded dataset (LeGrande & Schmidt, 2006). This approach gives us an interval for ocean water isotopic composition in the study area (last line in Table S1). The average isotopic composition of the ocean in this study is then assumed to be $\delta^{18}O_L$= 1.09‰ and δD_L = 7.25‰ (d-excess = -1.47).

Table S1: Isotopic composition and salinity for North Atlantic Ocean.

**Source**	**$\delta^{18}O$ ± 1SD (‰)**	**δD + 1SD (‰)**	**d-excess (‰)**	**Salinity (PSU)**	**Details**
BATS, 2012 (BIOS, 2021)	1.27 ± 0.07 [0.39 S – 13.1‰]	7.92 ± 0.81 [8.64 S – 307.6]	-2.22	36.55	BATS cruises: May – Sept 2012
Benetti et al. (2014)	1.24 ± 0.09 [0.28 S – 9.0‰]	8.18 ± 0.65 [1.58 S – 50.6‰]	-1.72	37.03	Strasse Cruise: Aug – Sept 2012 Eastern N. Atlantic
Benetti et al. (2017a)	1.19 ± 0.12 [0.32 S – 10.5‰]	8.03 ± 0.81 [2.04 S – 65.8‰]	-1.51	36.22	Rara Cruise: Mar and May 2015 Colibri Cruise: Aug 2016 Leg in Bermuda area
LeGrande and Schmidt (2006)	1.07 ± 0.15 [0.55 S – 18.98‰]	-	-	-	Averaged in a 10°x10° centered in Bermuda
Average in Bermuda Area	1.23	7.97	-0.51	36.39	BATS, 2012 Benetti et al. (2017a)
Estimated from δ vs S	1.12^{*} $1.06^{\dagger}$	8.29^{*} $6.20^{\dagger}$	-0.67^{*} $-2.28^{\dagger}$	36.32	June – Dec 2013

[δ vs S linear relationship]. δ vs S relationship for N. Atlantic (Benetti et al., 2017a): *Using δ vs S in for gridded dataset (LeGrande & Schmidt, 2006). †Using δ vs S estimated from *BIOS* (BIOS, 2021).

Text S2: Details on Craig Gordon (CG) model and non-equilibrium fractionation

The CG model can be parametrized into a five layers process, as shown in Figure S1, with an atmospheric component based on the Langmuir linear-resistance model for evaporation where transport of water molecules can be defined as a resistance to atmospheric transport. Starting from the bottom, the first layer is well-mixed ocean water. The second layer is the interface between water and air. Here, humidity is at saturation (h = 1) and equilibrium fractionation is assumed to be reached. The third layer is a diffusion-controlled layer where different water isotopologues are characterized by different diffusivities in air. The fourth layer is a turbulence-controlled layer. In this fourth layer, no isotopic fractionation occurs because all water isotopologues of water are characterized by identical mixing behavior. The fifth layer is the "Free atmosphere", where humidity and isotopologues profiles become less pronounced with height. The actual size of the layers 2-3-4 depends on location, time of the day and atmospheric conditions. However, it is expected that layers 2 and 3 are extremely thin and very hard to resolve with current measurement techniques (Madsen et al., 2019). Measurements above the ocean surface, like in this study, predominantly take place in the fifth or within the fourth layer (top and bottom inlet in Figure S1) making the abovementioned assumption "*The mixing process in the gradient measurement space is fully turbulent and does not introduce any fractionation*" reasonable.

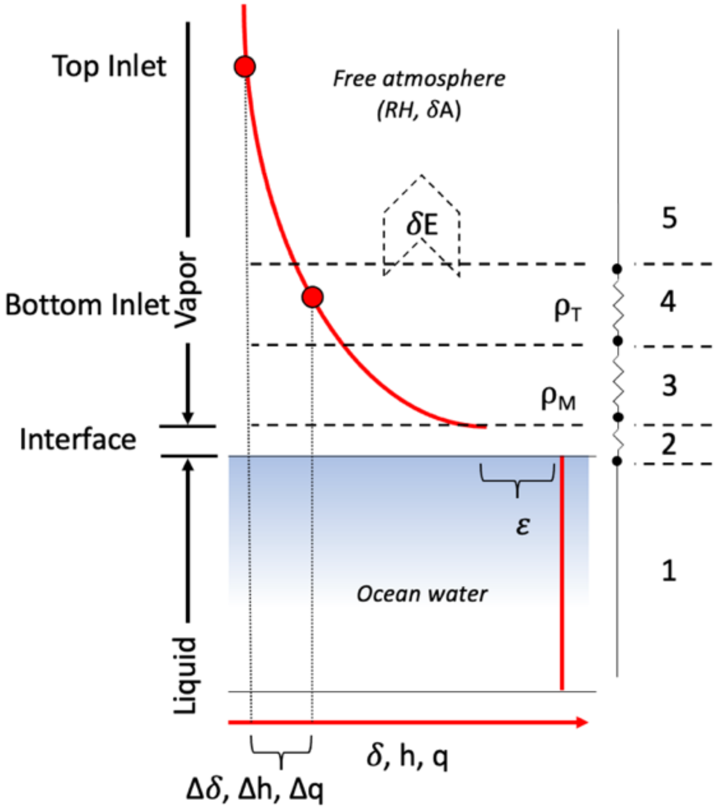

Figure S1: Visual representation of Craig-Gordon model with reference to measurement point of the THMAO tower. Layer size and sampling inlet heights are not to scale.

The non-equilibrium fractionation (k) depends on the ratio between turbulent and molecular transport and the diffusivities of different isotopologues in air, as defined in Merlivat and Jouzel, (1979):

$$\boldsymbol{k} = \frac{\left(\frac{D}{D_i}\right)^n - 1}{\left(\frac{D}{D_i}\right)^n + \frac{\rho_T}{\rho_M}} \qquad \text{(eq. S1)}$$

where the ratio D/D_i is the ratio between diffusivities of the rare isotopologue of water *i* and the common isotopologue of water; the exponent *n* depends on the wind regime and is equal to 2/3 and to 1/2 for smooth and rough wind regimes; ρ_T and ρ_M are the resistances relative to turbulent and molecular transport in air, respectively. In the Brutsaert evaporation model, the ratio of resistances can be estimated for smooth and rough surfaces with equations S2 and S3 (Brutsaert, 1965):

$$\frac{\rho_T}{\rho_M} = \frac{1}{\chi} \frac{ln\left(\frac{u_* z}{30\nu}\right)}{13.6\left(\frac{\nu}{D}\right)^n} \qquad \text{(eq. S2)}$$

$$\frac{\rho_T}{\rho_M} = \frac{1}{\chi} \frac{ln\left(\frac{z}{z_0}\right) - 5}{7.3 R_{es}^{1/4}\left(\frac{\nu}{D}\right)^n} \qquad \text{(eq. S3)}$$

where χ is Von Karman constant; u_* is the friction velocity [m s^{-1}]; ν is the kinematic air viscosity [kg m^{-1} s^{-1}]; *z* is the height above the water surface [m]; z_0 is the roughness length [m]; R_{es} is the surface roughness Reynolds number [-]. Therefore, the fractionation factor *k* can be directly calculated as a function of wind speed *u* [m s^{-1}] at a reference level (e.g. 10 m) because:

$$\boldsymbol{u}_* = \frac{\chi u}{ln\left(\frac{z}{z_0}\right)} \qquad \text{(eq. S4)}$$

$$\boldsymbol{R}_{es} = \frac{u_* z_0}{\nu} \qquad \text{(eq. S5)}$$

It has been proposed that equation S2 is valid when $R_{es} < 1$ and equation S3 when $R_{es} > 1$. This variation of transport regime produces a discontinuity in the *k* vs wind speed relationship. Such discontinuity for 10m wind speed is ≈ 6 m s^{-1}.

Text S3: Algorithm for choosing the best *k* value

Non-equilibrium fractionation factor *k* is estimated from a direct comparison between observed and modeled isotopic composition of evaporation flux. For a given flux observation *i*, it is possible to calculate *m* different values of flux composition with CG model by varying the kinetic fractionation factors within a certain range. Then, applying the same procedure to all the n-observations available, it is possible to obtain a *n x m* matrix that can be compared to observed flux in the following way:

$$\overline{\boldsymbol{DF}}_{(n\,x\,m)} = \left(\overline{\boldsymbol{CG}} - \overline{\boldsymbol{\delta}}_{\boldsymbol{E}}\right)^{\circ 2} \qquad \text{(eq. S6)}$$

where the matrix $\overline{\boldsymbol{\delta}}_{\boldsymbol{E}}$ is composed by the column vector of observed flux isotopic composition concatenated *m* times. Then, the differences between modeled and observed values are squared (Hadamard power) and the minimum difference for each observation can be calculated as follows:

$$\overline{\boldsymbol{min}}_{n\,x\,1} = \min_{j \in J}(\overline{\boldsymbol{DF}}), \ \forall \boldsymbol{i} \in \boldsymbol{I} \qquad \text{(eq. S7)}$$

being I [$1,\ldots,i,\ldots,n$] and J [$1,\ldots,j,\ldots,m$] the indices of the rows and columns of the matrix $\overline{\boldsymbol{DF}}$, respectively. Similarly, the vector of minimum differences can be concatenated m times to obtain the matrix $\overline{\boldsymbol{MIN}}_{\boldsymbol{n\,x\,m}}$ that can be compared with $\overline{\boldsymbol{DF}}$ to obtain a weighting matrix $\overline{\boldsymbol{BM}}$:

$$\overline{\boldsymbol{BM}}_{(\boldsymbol{n\,x\,m})} = \overline{\boldsymbol{DF}} == \overline{\boldsymbol{MIN}} \qquad \text{(eq. S8)}$$

where the double equal in equation S8 is used to distinguish Boolean equality comparison from variable assignation. The $\overline{\boldsymbol{BM}}$ matrix identify the elements of $\overline{\boldsymbol{CG}}$ that best match the observed isotopic composition of evaporation flux by minimization of the differences and, as a direct consequence, the best kinetic fractionation value for each observation ($\overline{\boldsymbol{BK}}_{\boldsymbol{n\,x\,1}}$). The mean kinetic fractionation value k then can be estimated with a weighted average using the error on flux composition ($\boldsymbol{\sigma E_i}$) as the weight:

$$\boldsymbol{k = \sum_{i=1}^{n} w_i * BK_i,} \qquad \boldsymbol{w_i} = \frac{\left|\frac{\sigma E_i}{\delta E_i}\right|}{\sum_{i=1}^{n}\left|\frac{\delta E_i}{\sigma E_i}\right|} \quad \text{(eq13)}$$

Text S4: Impact of data filtering on observations PDFs

After data filtering, top inlet $\delta^{18}O$ data distribution becomes less affected by depleted values maintaining approximately unchanged its mean and median values (mean = median ≈ -11‰ prior and after data filtering with a standard deviation of 1‰). The change in distribution shape is more appreciable for d-excess, where data filtering reduced the magnitude of secondary modes (see Figure S2 for $\delta^{18}O$ and d-excess).

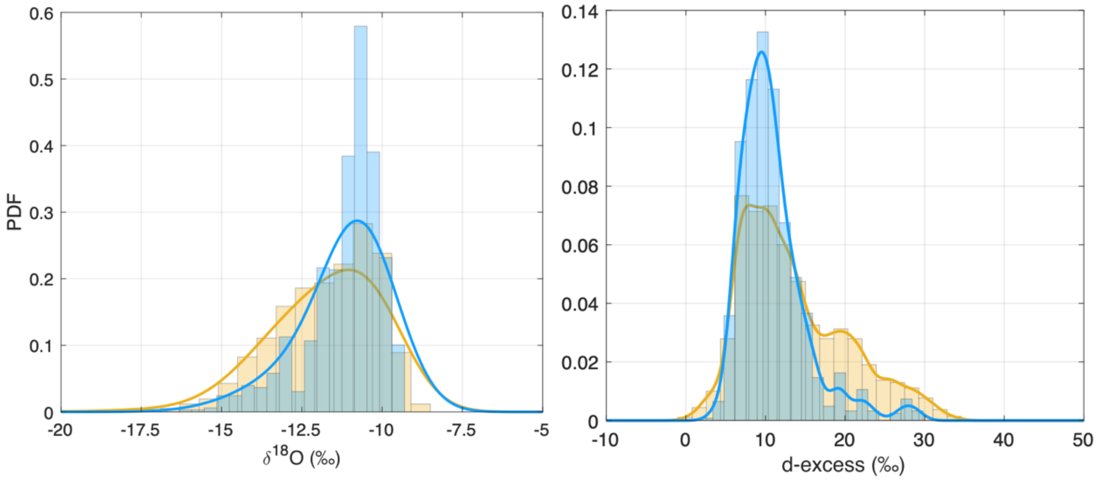

Figure S2: Distribution of water vapor isotopic composition prior (orange) and after data filtering (blue) for Top inlet. A continuous kernel density function was estimated with bandwidth = 1‰.

Presence of several modes in d-excess can be attributed to concurrence of different process involved in water vapor d-excess signal such as change in moisture source area, rain droplets evaporation and increased influence of entrainment. After data filtering, d-excess mean ± 1 standard deviation lowered from 15 ± 6 ‰ to 12 ± 4 ‰.

Correlation between water vapor d-excess and h (RH relative to SST) is very high in Bermuda (R=-0.91, for all top inlet observations). However, dataset reduction lowered the linear regression coefficients significantly, from -0.48 ‰/% to -0.46 ‰/% and from 48 ‰ to 46 ‰, for slope and

intercept, respectively (Figure S3). The slope (intercept) increases (decreases) significantly more when the dataset is filtered by removing observations with boundary layer height (BLH) e.g. larger than 1000 m: -0.39 ‰/% (0.40‰)

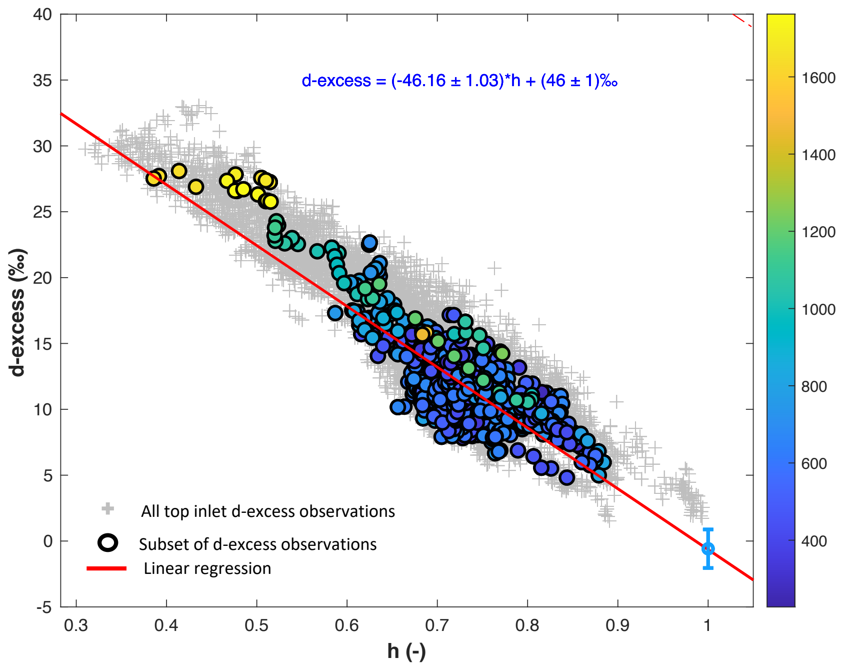

Figure S3: Relationship between d-excess, *h* and boundary layer height (BLH, color index). Colored circles represent reduced dataset observations from the top inlet observations after data reduction. D-excess of water vapor in isotopic equilibrium with ocean water is -0.8 ± 0.7‰, estimated with ocean water composition $\delta^{18}O_L$ = 1.09 ± 0.03‰, δD_L =7.25 ± 1.05‰ and mean SST = 25.9°C. Linear regression was calculated for reduced data (R^2 = 0.70).

The linear regression model between h and d-excess is able to reproduce the estimated d-excess of water vapor in isotopic equilibrium with ocean water. The predicted d-excess at h=1 resulted -0.31‰ and -0.34‰ for full and filtered dataset, respectively. Previous long-term water vapor observations in Bermuda showed that variability on regression parameters is linked to season and to wind direction (Steen-Larsen et al., 2015). Moreover, as recently pointed out in (Benetti et al., 2018), mixing with free tropospheric air above the boundary layer plays an important role in water vapor d-excess variability in the North Atlantic. Bermuda d-excess is highly correlated with BLH (R=0.74), and the correlation is still high after data reduction (R=0.63).

Typical fully developed boundary layer height is 800 m at 15:00 (LST) which tends to increase from November and later on (1100 m, centered between 12:00 and 15:00 LST). However, the number of observations characterized by large BLH values (> 1100 m) was significantly reduced after data filtering (Figure S4), from ~17% to ~4%.

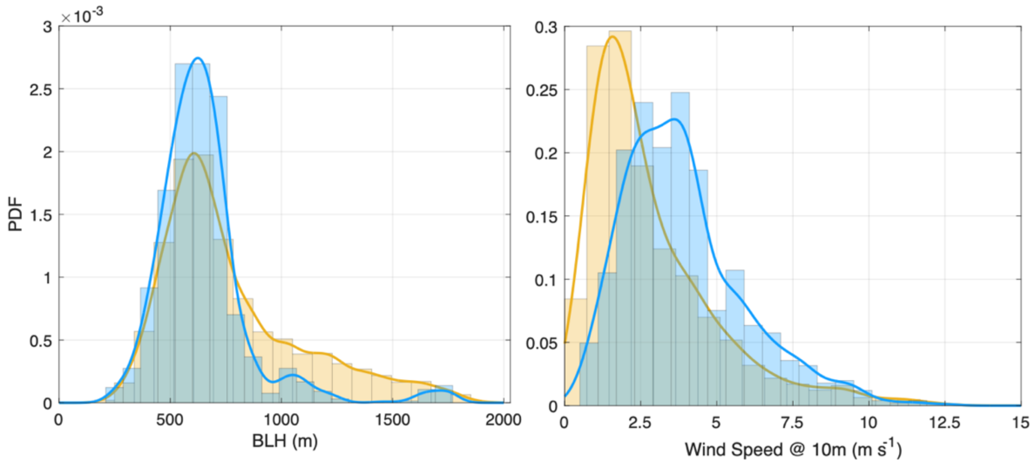

Figure S4: Distribution of BLH and WS datasets prior (orange) and after data reduction (blue). A continuous kernel density function was estimated with bandwidth = 50 m and 0.5 ms^{-1} for BLH and WS, respectively. WS was corrected to 10m height.

The main mode, the mean, and the median of BLH does not change significantly after data filtering (from 607 m, 777 m, 679 m to 623 m, 646 m, 620 m for mode, mean and median, respectively). Bottom and top inlet heights are within 1/10 of BLH in 80% of the cases. Therefore, most of the observations can be assumed to be performed within the surface layer, assuming the surface layer height to be roughly the bottom 10% of the BLH (Geernaert, 2003). On the contrary, data filtering significantly affected the wind speed distribution at the study site, largely reducing the number of observations characterized by low wind speed. The distribution shape after data reduction cannot still be considered of the normal-type but still of the Weibull-type. However, skewness reduced from 1.6 to 0.96 with mode, mean and median of WS changed from 1.6 ms^{-1}, 2.8 ms^{-1}, 2.2 ms^{-1} to 3.6 ms^{-1}, 4.0 ms^{-1}, 3.7 ms^{-1}. Therefore, the main consequence of data reduction from the perspective of d-excess sensitivity to ocean surface condition is a larger impact of shallow mixing with lesser influence on large marine boundary layer development and on low wind speed conditions.

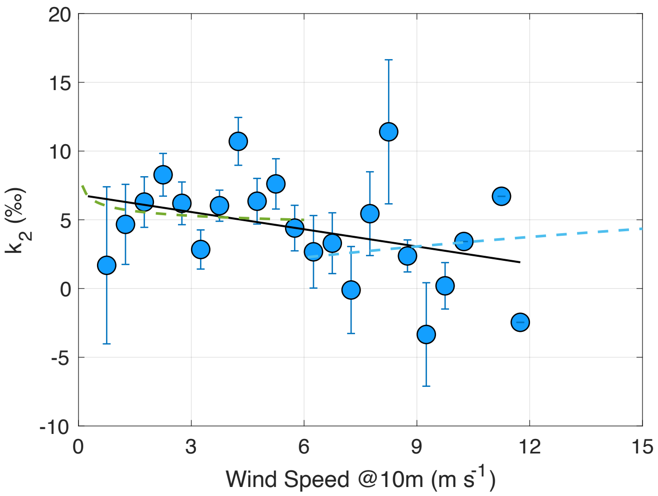

Figure S5: Mean k_2 values obtained for each wind speed class. Dashed lines represent the non-equilibrium fractionation factor parametrization as a function of WS at 10m height. Solid black line represents a linear fit $k_2 = (-0.4 \pm 0.3) *WS + (7 \pm 2)$ ‰ (R^2=0.12) in the wind speed interval 0 – 10 m s^{-1}.

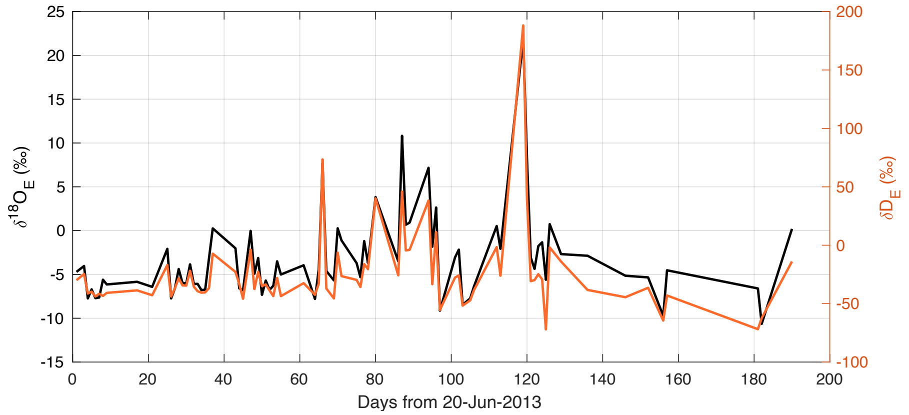

Figure S6: Timeseries of isotopic composition of the evaporation flux as estimated from KP intercept.

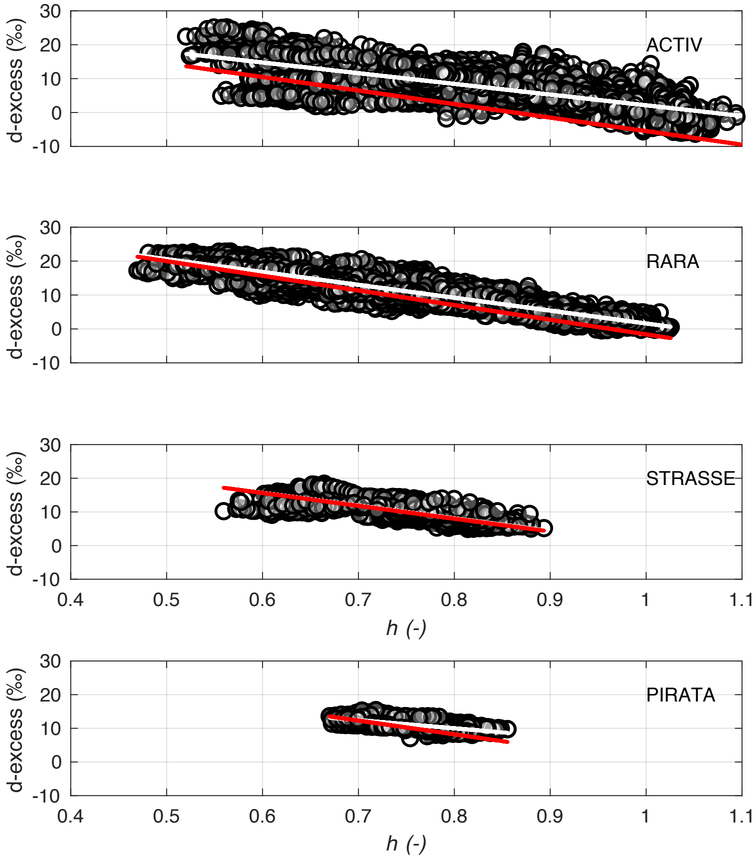

Figure S7: Relationship between d-excess and h_s for cruises reported in Benetti et al. (2017b). Observations averaged over 15 minutes are depicted as circles. The white line is a fit of observed d-excess vs h_s. The red line is a fit of modeled d-excess vs h_s using the closure assumption (k_{18}=5.2‰, k_2=4.3‰).

References

Benetti, M., Reverdin, G., Pierre, C., Merlivat, L., Risi, C., Steen-larsen, H. C., & Vimeux, F. (2014). Deuterium excess in marine water vapor: Dependency on relative humidity and surface wind speed during evaporation. *Journal of Geophysical Research: Atmospheres*, *119,* 584–593. https://doi.org/10.1002/2013JD020535

Benetti, M., Reverdin, G., Aloisi, G., & Sveinbjörnsdóttir, Á. (2017a). Stable isotopes in surface waters of the A tlantic Ocean: Indicators of ocean-atmosphere water fluxes and oceanic mixing processes. *Journal of Geophysical Research: Oceans*, *122*(6), 4723–4742. https://doi.org/10.1002/2017JC012712

Benetti, M., Steen-Larsen, H. C., Reverdin, G., Sveinbjörnsdóttir, Á. E., Aloisi, G., Berkelhammer, M. B., et al. (2017b). Data Descriptor: Stable isotopes in the atmospheric marine boundary layer water vapour over the Atlantic Ocean, 2012-2015. *Scientific Data*, *4*, 1–17. https://doi.org/10.1038/sdata.2016.128

Benetti, M., Lacour, J. L., Sveinbjörnsdóttir, A. E., Aloisi, G., Reverdin, G., Risi, C., et al. (2018). A Framework to Study Mixing Processes in the Marine Boundary Layer Using Water Vapor Isotope Measurements. *Geophysical Research Letters*, *45*(5), 2524–2532. https://doi.org/10.1002/2018GL077167

BIOS. (2021). Bermuda Atlantic Time-series Study (BATS). http://bats.bios.edu/ (Last accessed on: 14/05/2021).

Brutsaert, W. (1965). A model for evaporation as a molecular diffusion process into a turbulent atmosphere. *Journal of Geophysical Research*, *70*(20), 5017–5024. https://doi.org/10.1029/jz070i020p05017

Geernaert, G. L. (2003). Boundary Layers | Surface Layer. *Encyclopedia of Atmospheric Sciences*, (1988), 305–311. https://doi.org/10.1016/b0-12-227090-8/00092-0

LeGrande, A. N., & Schmidt, G. A. (2006). Global gridded data set of the oxygen isotopic composition in seawater. *Geophysical Research Letters*, *33*(12), 1–5. https://doi.org/10.1029/2006GL026011

Madsen, M. V., Steen-Larsen, H. C., Hörhold, M., Box, J., Berben, S. M. P., Capron, E., et al. (2019). Evidence of Isotopic Fractionation During Vapor Exchange Between the Atmosphere and the Snow Surface in Greenland. *Journal of Geophysical Research: Atmospheres*, 124(6), 2932–2945. https://doi.org/10.1029/2018JD029619

Merlivat, L., & Jouzel, J. (1979). Global climatic interpretation of the deuterium-oxygen 18 relationship for precipitation. *Journal of Geophysical Research: Oceans*, *84*(C8), 5029–5033. https://doi.org/10.1029/JC084iC08p05029

Steen-Larsen, H. C., Sveinbjörnsdottir, A. E., Jonsson, T., Ritter, F., Bonne, J. -L., Masson-Delmotte, V., et al. (2015). Moisture sources and synoptic to seasonal variability of North Atlantic water vapor isotopic composition. *Journal of Geophysical Research Atmospheres*, *120*. https://doi.org/10.1002/2015JD023234
